# Opioid Exposure Measurement in Postacute Care Under Medicare Consolidated Payments

**DOI:** 10.1001/jamahealthforum.2025.3724

**Published:** 2025-09-12

**Authors:** Kelsey L. Corcoran, Kaleen N. Hayes, Richa Joshi, Sarah D. Berry, Andrew R. Zullo

**Affiliations:** 1 Center for Gerontology and Healthcare Research, Brown University School of Public Health, Providence, Rhode Island; 2 Department of Health Services, Policy, and Practice, Brown University School of Public Health, Providence, Rhode Island; 3 Department of Epidemiology, Brown University School of Public Health, Providence, Rhode Island; 4 Hinda and Arthur Marcus Institute for Aging Research, Hebrew SeniorLife and Harvard Medical School, Boston, Massachusetts; 5 Department of Medicine, Beth Israel Deaconess Medical Center, Harvard Medical School, Boston, Massachusetts

## Abstract

This cohort study quantifies the extent to which sole reliance on Medicare Part D claims may underestimate medication dispensing using opioids after hip fracture hospitalization.

## Introduction

During hospital and posthospital skilled nursing facility (SNF) care, medications are bundled into Medicare Part A consolidated payments leaving medications unmeasured in the Centers for Medicare & Medicaid (CMS) data.^1^ Medication changes occurring in this period are not reflected in Part D claims. We quantify the extent to which sole reliance on Part D claims underestimates medication dispensing using opioids after hip fracture hospitalization as an example.

## Methods

This cohort study used 2012-2018 data from Omnicare, a long-term care pharmacy (LTCP), for Medicare fee-for-service (FFS) beneficiaries hospitalized for a hip fracture before SNF admission, with no opioid dispensing the year prior. We linked the LTCP data to Part D claims (eMethods in Supplement 1) then examined the concordance between LTCP and Part D measures of opioid dispensing for 100 days following SNF admission. All dispensed regimens were included. Opioid persistence was calculated using days’ supply on day 31 after SNF admission.^2^ The study was approved by the research ethics board of Brown University, including a waiver of patient consent as deidentified data. The STROBE reporting guideline was followed. Data were analyzed from March 19, 2024, to July 9, 2025, with SAS version 9.4 (SAS Institute Inc).

## Results

There were 52 586 opioid-naive Medicare beneficiaries discharged to SNFs after hip fracture hospitalization. The mean (SD) age was 85.7 (7.7) years, 75.9% were female and 24.1% were male. The mean (SD) length of SNF stay was 47.9 (28.7) days.

**Figure 1.**
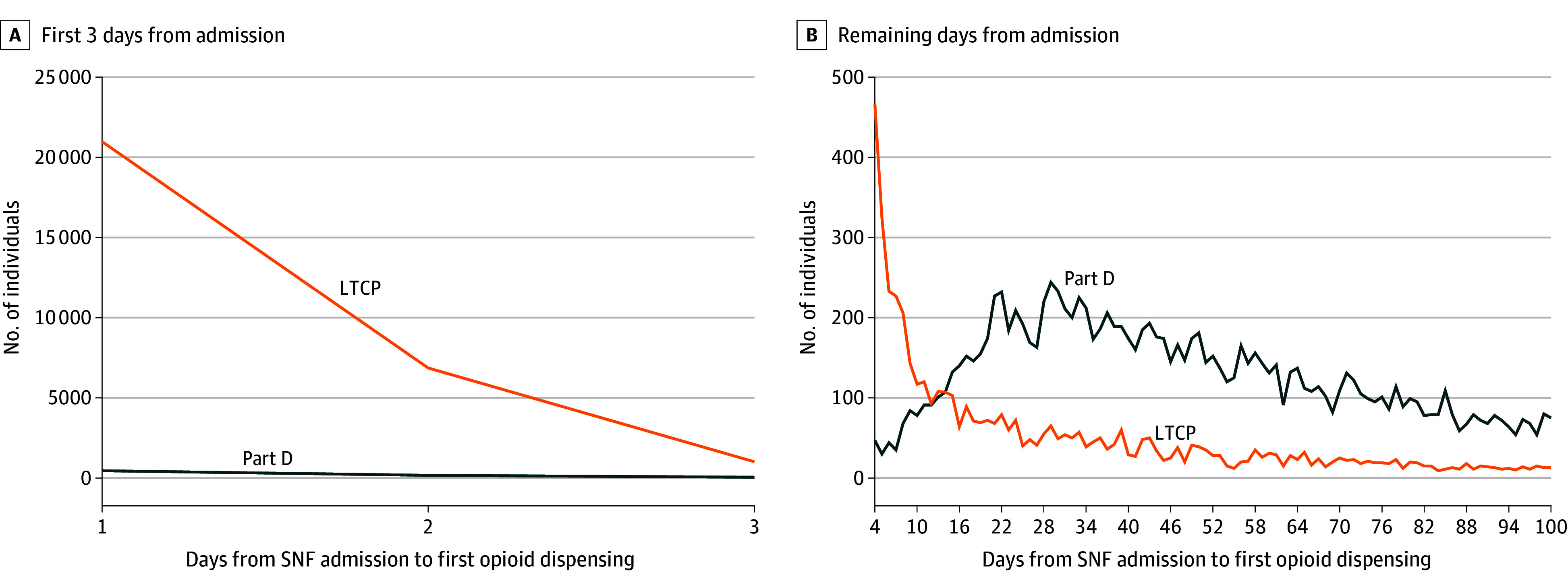
Time to Opioid Dispensing in Skilled Nursing Facilities (SNFs) Following Hip Fracture in Part D vs Long-Term Care Pharmacy (LTCP) Data

Overall, 71.4% of the sample received opioids within 100 days of SNF admission. The median time to opioid dispensing from SNF admission was 1 (IQR, 1-2) day in the LTCP data and 42 (IQR, 25-63) days in Part D claims (Figure 1). Of 33 509 individuals who received opioids within 30 days of SNF admission, 34.7% used opioids persistently on day 31 (86.5% in LTCP data, 19.4% in Part D).

**Figure 2.**
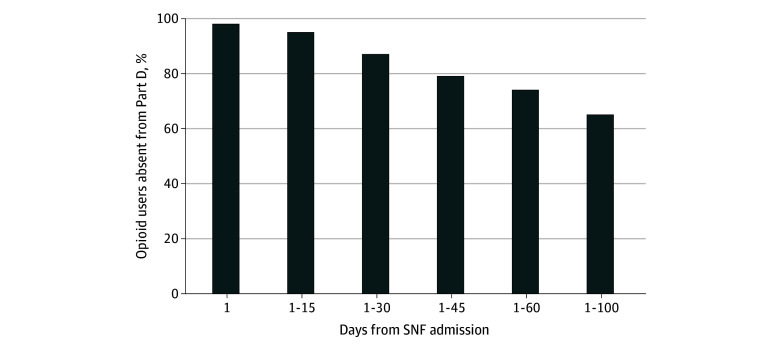
Percent of Opioid Users Absent From Part D Among Skilled Nursing Facility (SNF)–Admitted Individuals Following Hip Fracture Hospitalization

On the day of SNF admission, 98% of individuals who received opioids would have been classified as unexposed to opioids if using Part D data alone (Figure 2). By 100 days, 65% of opioid-exposed individuals were absent from Part D data.

## Discussion

These findings reflect the constraints of Medicare claims-based medication measurement due to billing structures. A limitation of this study is that it uses data from a single national LTCP serving only a subset of US SNFs. While short durations of opioids for postacute care are consistent with clinical expectations, this unsurprisingly increases the likelihood that opioid exposure is captured by LTCP data and absent from Part D claims.

Investigators using CMS data should be cautious when identifying medication use if their study period includes hospital or SNF stays, as this illustrates the broader challenge that Part D data can be misinterpreted without an understanding of Medicare’s billing structure. Projections of opioid prescribing, access to nonopioid pharmacologic and nonpharmacologic pain treatments, and opioid use disorder therapies may be affected. The potential underestimation of Medicare FFS beneficiaries’ medication use likely varies across medication classes and health conditions and may have implications for research, policy, and program implementation.

Annually, 1.4 million Medicare FFS beneficiaries will require an SNF stay,^3^ which is disproportionately common among vulnerable populations, including those with Alzheimer disease and related dementias, mental illness, and preventable readmissions.^3,4,5^ Without data on medications during SNF stays, it will be exceedingly challenging to advance research efforts to understand pharmacotherapy and its outcomes in postacute SNF care. We believe CMS could take steps toward facilitating critically necessary science by entering into agreements with LTCPs to obtain medication data during SNF stays. This may be feasible since most of the SNF market is served by just 2 pharmacies (Omnicare and PharMerica).^6^

## Notes

Supplement 1. **eMethods.** Data Linkage

Supplement 2.
Data Sharing Statement
