# Supplementary material for: Opioid Exposure Measurement in Postacute Care Under Medicare Consolidated Payments
Source: JAMA Health Forum. 2025 Sep 12;6(9):e253724. doi: 10.1001/jamahealthforum.2025.3724 (PMC12432634; doi:10.1001/jamahealthforum.2025.3724)
Supplement: Supplement 1. — eMethods. Data Linkage [file jamahealthforum-e253724-s001.pdf]

Supplemental Online Content

Corcoran KL, Hayes KN, Joshi R, Berry SD, Zullo AR. Opioid exposure measurement in postacute care under Medicare consolidated payments. *JAMA Health Forum.* Published online September 12, 2025. doi:10.1001/jamahealthforum.2025.3724

eMethods. Data Linkage

This supplemental material has been provided by the authors to give readers additional information about their work.

© 2025 Corcoran KL- et al. *JAMA Health Forum*.

eMethods. Data Linkage

Linkage occurred in a multi-step process. First, individuals with a record in the Omnicare long-term care pharmacy data (2012-2018) were matched deterministically to Medicare beneficiary records using first name, last name, date of birth, and sex. Deterministic linkage was conducted by General Dynamics Information Technology (GDIT), creating a crosswalk of unique, encrypted identifiers by which to link eligible Medicare beneficiaries to the Omnicare records. Of 4,934,037 eligible individuals in Omnicare, 3,453,836 (70%) were successfully matched on all four dimensions to a Medicare beneficiary. Next, the list of matched beneficiaries was restricted to a finalized cohort of 52,586 unique opioid-naïve Medicare beneficiaries who were hospitalized for hip fracture, created through the following steps:

1) We first identified all Medicare beneficiaries with a principal diagnosis of hip fracture on an inpatient hospital record and then subsequently discharged to a skilled nursing facility (SNF) for post-acute care between 01/01/2012 and 12/31/2018, sampling the first eligible hip fracture hospitalization during the study period.
2) We then restricted to beneficiaries who were aged ≥65 years as of the hospital admission.
3) We then excluded those who were not continuously enrolled in Medicare Parts A, B, and D for at least 12 months prior to their hip fracture hospitalization; resided outside of the 48 contiguous states; or died during the hospitalization. We also excluded individuals who died, disenrolled from Medicare, and were dispensed opioids in the year prior to SNF admission.

© 2025 Corcoran KL- et al. *JAMA Health Forum*.
