# Supplementary material for: Opioid Exposure Measurement in Postacute Care Under Medicare Consolidated Payments
Source: JAMA Health Forum. 2025 Sep 12;6(9):e253724. doi: 10.1001/jamahealthforum.2025.3724 (PMC12432634; doi:10.1001/jamahealthforum.2025.3724)
Supplement: Supplement 2. — Data Sharing Statement [file jamahealthforum-e253724-s002.pdf]

Data Sharing Statement

Corcoran. Opioid Exposure Measurement in Postacute Care Under Medicare Consolidated Payments. *JAMA Health Forum*. Published September 12, 2025. doi:10.1001/jamahealthforum.2025.3724

Data
Data available: No

Additional Information
Explanation for why data not available: Use of data from the Centers for Medicare & Medicaid Services (CMS) and Omnicare was covered under the strict terms of a Data Use Agreement (DUA) and individual-level data cannot be shared. Researchers seeking access to CMS data for their own studies should visit the Research Data Assistance Center (ResDAC) at www.resdac.org to get started.
